# RS-SSKD: Self-Supervision Equipped with Knowledge Distillation for Few-Shot Remote Sensing Scene Classification

**DOI:** 10.3390/s21051566

**Published:** 2021-02-24

**Authors:** Pei Zhang, Ying Li, Dong Wang, Jiyue Wang

**Affiliations:** 1 School of Computer Science, National Engineering Laboratory for Integrated Aero-Space-Ground-Ocean Big Data Application Technology, Shaanxi Provincial Key Laboratory of Speech & Image Information Processing, Northwestern Polytechnical University, Xi’an 710129, China; cszhangpei@mail.nwpu.edu.cn (P.Z.); dongwang@mail.nwpu.edu.cn (D.W.); 2 School of Electronic and Information Engineering, South China University of Technology, Guangzhou 510641, China; wang.jiyue@mail.scut.edu.cn

**Keywords:** remote-sensing, scene classification, few-shot learning, meta-learning, self-supervised, knowledge distillation

## Abstract

While growing instruments generate more and more airborne or satellite images, the bottleneck in remote sensing (RS) scene classification has shifted from data limits toward a lack of ground truth samples. There are still many challenges when we are facing unknown environments, especially those with insufficient training data. Few-shot classification offers a different picture under the umbrella of meta-learning: digging rich knowledge from a few data are possible. In this work, we propose a method named RS-SSKD for few-shot RS scene classification from a perspective of generating powerful representation for the downstream meta-learner. Firstly, we propose a novel two-branch network that takes three pairs of original-transformed images as inputs and incorporates Class Activation Maps (CAMs) to drive the network mining, the most relevant category-specific region. This strategy ensures that the network generates discriminative embeddings. Secondly, we set a round of self-knowledge distillation to prevent overfitting and boost the performance. Our experiments show that the proposed method surpasses current state-of-the-art approaches on two challenging RS scene datasets: NWPU-RESISC45 and RSD46-WHU. Finally, we conduct various ablation experiments to investigate the effect of each component of the proposed method and analyze the training time of state-of-the-art methods and ours.

## 1. Introduction

Scene classification is one of the most fundamental tasks in the remote sensing community, it plays a vital role in semantic understanding of remote sensing (RS) scenes. In addition, it provides significant support for various important applications and societal needs, including urban planning [1], land-cover analysis [2], environmental monitoring [3], deforestation mapping [4], air pollution prediction [5], etc. In computer vision, image-level classification has been marked by extraordinary progress in the last few years. Much of this progress has come from deep learning since the emergence of the AlexNet [6] in 2012. The convolutional neural network (CNN) has continued to dominate in the following years and has recently achieved human-level performance on certain image classification benchmarks [7,8,9]. In the remote sensing community, RS scene classification has been well studied for the last few decades. The methods using handcrafted features [10,11,12,13,14,15,16,17] have been the leading approach in earlier years; however, they require hand design features and lack adaptability. This method family performs poorly for complex scenes or massive data and has been replaced by deep learning methods.

Now, the state-of-the-art approaches to RS scene classification [18,19,20,21,22,23,24,25] are mainly CNN based models. These methods aim to automatically learn global features from the input data using deep convolutional neural networks (e.g., AlexNet [6], VGGNet [7], GoogLeNet [26], and ResNet [9]), which generates a high-level representation useful to classify RS scene images.

One approach of the previous work using CNN for RS scene classification takes the transfer learning strategy: pre-train on other large datasets then fine-tuning on RS scene datasets. It is the case that Hu et al. [18] proposed two strategies for transferring features from pre-trained CNNs on ImageNet [8]. The first strategy is directly extracting features from the FC layers, while the latter encodes multi-scale dense features extracting from the last convolutional layer into global image features. Their methods have very much defeated the traditional handcrafted methods [15,16,17] on the UC Merced Land-Use (UCM) dataset [16] and WHU-RS [27] dataset. Similarly, three learning strategies (i.e., full training, fine-tuning, and using pre-trained CNNs as feature extractors) are proposed in the literature [20]. Six popular CNNs are exploited in their experiments in three remote sensing datasets, namely the UCM dataset [16], RS19 dataset [27], and the Brazilian Coffee Scenes dataset [28].

Another stream of approach is devoted to improve the structure of existing CNN networks or modifying the loss function. Wang et al. [29] presented an improved oriented response network by adding active rotating filters into the architecture. Besides that, a squeeze layer is injected before the align operation to help extract the orientation descriptors. Cheng et al. [19] proposed a novel metric learning regularization term beyond the normal cross-entropy loss to address the problem of within-class diversity and between-class similarity. Considering the importance of feature embedding and metric space, Kang et al. [25] developed a method to improve RS scene discrimination by combining two components. First, the author introduced a joint loss function that takes advantage of both SNCA [30] loss and cross-entropy loss to tackle the within-class diversity and between-class similarity inherent to RS scenes. Then, a novel optimization mechanism based on momentum update is utilized for minimizing the joint loss function.

Despite these and other state-of-the-art methods have made significant progress to date and have even achieved ∼100% accuracy on some datasets (e.g., [24] achieves 99.82% accuracy on the UC Merced dataset [16,31] achieves 99.46% on the WHU-RS19 dataset [27]), one may argue, is that machine learning really outperforming human performance, or is the dataset too simple? For example, the UC Merced dataset holds 21 scene classes with 100 images per class, while the WHU-RS19 dataset contains 19 classes with ∼50 images in each. Can such a small scale of scene classes represent the scenarios of our real-world? Can such limited images per class represent the scene variations and diversity? An intuitive way to address this issue is to extend and enrich these datasets. Many researchers have begun to collect and label more data; significant efforts have been dedicated to constructing more massive RS scene datasets, e.g., the AID dataset [32],the NWPU-RESISC45 dataset [22], the PatternNet dataset [33], and the RSD46-WHU dataset [34]. Publicly available RS scene datasets are summarized in Table 1.

Although the RS scene datasets are expanding in scale, they are still considered small from the perspective of deep learning, which requires large amounts of training data. Meanwhile, a different picture has emerged in the machine learning area, highlighting the significance of digging “rich” knowledge from “a few” data. For example, when dealing with bio-information or drug discovery [36], collecting supervised information is highly time- and cost-intensive. For a robot, it must learn quickly and efficiently in a complex and ever-changing environment. If it is able to learn from one-shot human demonstration that would be a massive advance in general intelligence [37]. People can comprehend a novel scene (e.g., shared-bike parking lot) from just one, or a handful examples, while a neural network model has to solve the task from scratch. These needs drive us to develop human-like learning and thinking models. Toward this goal, researchers proposed a challenging setting: few-shot learning (FSL) [38,39,40].

**Figure 1:**
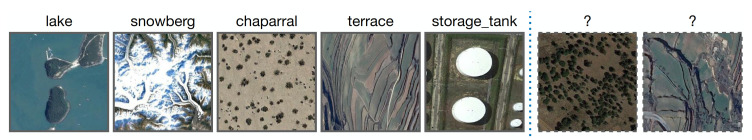
The One-shot Challenge: few-shot learning from one example. A single example of a new visual scene can be enough information for a child to classify new examples.

FSL intends to learn a model that can quickly generalize to new tasks from very few training examples. This is at odds with previous studies in the machine learning field: from the statistical machine learning standpoint, enough training examples are necessary to reveal the data distribution, ensuring that the model is learnable and generalizable. One might wonder how humans have the impressive ability to generalize or infer from only a few or even one image? Strong prior knowledge and experience must be a critical discrepancy between human and Artificial Intelligence (AI). For example, ask a young child to classify new scenes like chaparral or terrace that he/she has never seen before, with just one instance per class for a glance. In addition, there is a high probability that the child will be able to give the correct answer. It is a case showing that humans can rapidly adapt to a new task based on their previous knowledge learned from related tasks, shown as Figure 1.

Few-shot learning suggests that a human-like learning paradigm where a model gains common knowledge across a set of tasks often derives from the same distribution of related tasks and employs this knowledge to enhance its future learning performance [38]. Concretely, few-shot classification is one of the most well-studied test-bed for FSL, which aims to learn a model on SEEN categories and perform classification on new categories (UNSEEN) with only a limited amount of labeled training examples. To this end, prior work has suggested acquiring cross-task knowledge (meta-knowledge) and rapid learning ability through the manner of meta-learning [41]. *Learning to learn* [42,43,44] and *learning to compare* [38,40,45,46] can all be regarded as meta-learning. We will introduce the related work in Section 2.

Thus far, only a few efforts in remote sensing have focused on the few-shot classification topic. A well-known algorithm, Model-agnostic meta-learning (MAML) [42], is evaluated for few-shot problems in land cover classification [47]. The work [48] brings few-shot learning into the RS scene classification by combining pre-trained CNN and ProtoNet. Li et al. [49] introduce an approach based on Protypical Networks (ProtoNet) [40] to explore RS scene few-shot classification. The authors of [50] provide a testbed for few-shot classification of RS scene and re-implemented several well-known few-shot learning approaches with a deeper backbone Resnet-12 for a fair comparison. While many existing FSL models [45,51,52] focus on devising different architectures, we argue that feature embedding are overlooked. Well-learned representations may be more potent for few-shot classification than the prevailing complicated meta-learning algorithms. In this paper, our vision is to learn a powerful embedding, without any additional annotation effort that offers more efficient and effective representations to downstream meta-learner. To this end, we propose a method named RS-SSKD to solve the few-shot RS scene classification problem in the real world. In summary, our key contributions are:

- We propose a novel method, RS-SSKD, which provides powerful embeddings for the downstream meta-learning module. To achieve that, we design a Self-supervised Knowledge Distillation (SSKD) module that incorporates two different components: (1) a self-supervised network improves feature embedding, and (2) a knowledge distillation procedure boosts performance.
- We propose a self-supervised network with two identical branches that takes three pairs of original-transformed images as inputs. It enables the network to locate the category-specific relevant regions in the image and reduce the distraction caused by irrelevant parts, which guarantees the network generates discriminative embeddings.
- Building upon the self-supervised network, we utilize self-knowledge distillation to retrain the predictions of the trained model as new target values, which further boost the model’s performance.
- We evaluate the proposed method on two challenging RS scene datasets, where it achieves state-of-the-art (SOTA) performances compared with previous few-shot learning approaches. We also conduct various ablation experiments to verify the effectiveness of each component of the SSKD module.

The rest of this paper is organized as follows. We start with a brief review of the few-shot learning (FSL) literature in Section 2, and then we introduce the background and notations of the FSL problem in Section 3. The proposed method is stated in Section 4. In Section 5, we carry out extensive experiments on two RS scene datasets. Finally, Section 6 concludes this work and points out interesting further research.

## 2. Related Work

Human learners can learn a new concept from just one example; rather than learning from scratch, they are armed with previous knowledge [53]. Transfer learning is once a successful story by adopting an intuitive idea that fine-tunes a pre-trained model to utilize prior experience. However, its performance is poor when fine-tuning with only one or a few examples. A more challenging setting is proposed, i.e., few-shot learning (FSL) [38,39,40], which aims to learn a model from the SEEN categories in the base-set that can be quickly generalized to the UNSEEN categories in the novel-set under a limited data budget, usually 1 or 5 support samples. The literature on few-shot classification is vast; we summarized here briefly by two main streams: *learning to learn* and *learning to compare*.

**Learning to learn.** This family of approaches is often viewed as the most typical meta-learning, which refers to learn a general-purpose model that can be improved over multiple learning tasks. Each new task is expected to be learned better than the last, such that the model can generalize quickly to a new task. The most well-known of this group is perhaps MAML [42], which aims to learn a proper initialization of the model parameters. The intuition is to find certain model parameters that are more sensitive to changes in the task, so that small changes to these parameters will yield massive improvements on the loss function. Many variants of MAML follows this idea. Reptile [43] simplifies the meta parameters updating procedure by randomly sampling a task and performing K steps of SGD on it. LEO [44] introduces a task-dependent latent embedding space, in which the parameters of each task are initialized conditioning on the input data; such a strategy leads to more effective adaptation.

**Learning to compare.** The core idea of learning to compare is mapping the input to a feature space suitable for comparison and learning a task-dependent metric. It is also known as a metric-based approach. Matching Networks [38] learns different embedding functions for support and query examples and adopts an attention kernel to predict the query sample label. Prototypical networks (ProtoNet) [40] is based on a simple idea of comparing query instances with category prototypes. A prototype is defined as the mean embedding of the support examples. Much of the subsequent work [45,46] has been inspired by ProtoNet, which employ different metrics to classify query samples by the distance to prototype representations. TADAM [51] boosts the performance of ProtoNets by metric scaling, task-conditioning, and auxiliary task co-training. MetaOptNet [52] and FEAT [54] follow the same spirit of learning task-specific embeddings to ensure the features are more discriminative for a given task.

Recently, another line of work has begun to focus on learning richer representations. Zhao et al. [55] proposed a multitask learning framework that combines self-supervised learning and scene classification via a mixup loss strategy that enforces the network to learn more discriminative features without increasing the parameters. Benefiting from the preprocessing of the differential morphological profiles, the work [56] reduces the computation when using differential morphological profiles for classification, which requires relatively few features while achieving the same accuracy. A classification method [57] based on multi-structure deep features fusion (MSDFF) provides another perspective in which the complementarity of features extracted by different CNNs can capture deep features from the image. As pointed out in [58], it remains a discussion of whether rapid learning or feature reuse will lead to state-of-the-art performance. The authors of [58] analyzed MAML [42] and found that feature reuse is the dominant component in MAML’s effectiveness. Inspired by this suggestion, we propose a self-supervised knowledge distillation (SSKD) module that strives to learn a powerful embedding for the downstream meta learner.

## 3. Preliminary

**Figure 2:**
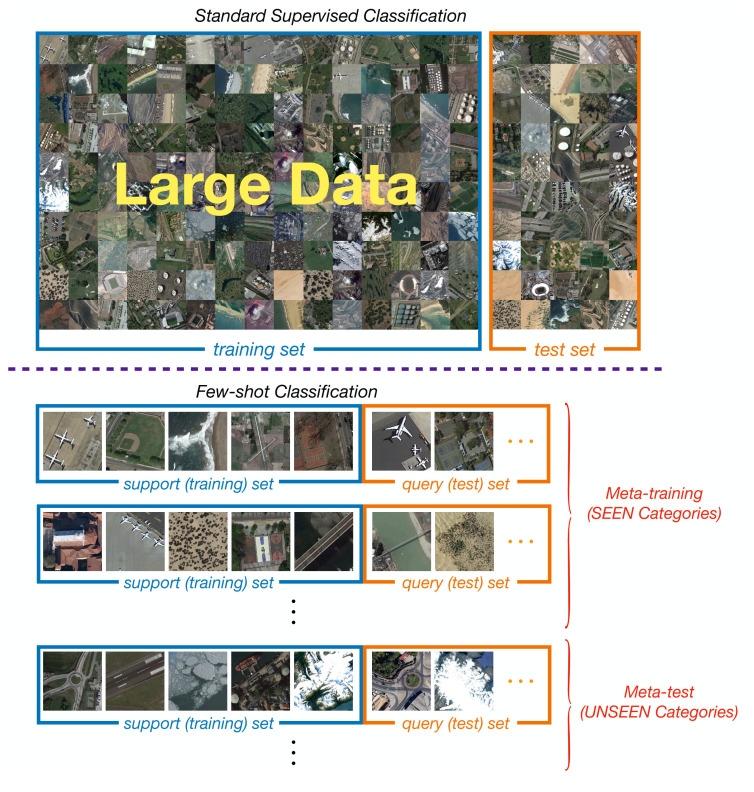
Comparison between the standard supervised classification and few-shot classification. The top represents the standard supervised classification mechanism in which a model is trained on a large dataset. The bottom shows a *task/episode* paradigm in FSL where we are dealing with the 1-shot, 5-way classification task.

Before presenting the main flow of our method in detail, we first introduce the definition and key notations of the Few-Shot Learning (FSL) setting, as the background of FSL might be new to some readers. The comparison between standard supervised classification and the few-shot learning paradigm is shown in Figure 2.

In a standard supervised learning setting, we are interested in a dataset $D=\left\{D_{train},D_{test}\right\}$. A model is trained on the $D_{train}$ with enough labeled data and is evaluated on the test set $D_{test}$. In few-shot learning (FSL) setting, we are given a meta dataset D, divided into $D_{base}$, $D_{val}$, and $D_{novel}$ by categories. Let C denote the category, $C_{base}$, $C_{val}$, and $C_{novel}$ are chosen to be mutually disjoint. The goal is to learn a model on $D_{base}$ with SEEN categories, which can generalize quickly on the UNSEEN categories in $D_{novel}$ when providing limited labeled examples. Note that an extra dataset-split $D_{val}$ is held out for selecting the hyper-parameter and choosing the best model.

Unlike standard machine learning trains over the instance level, Vinyals et al. [38] suggest a *task/episode* strategy to learn a meta-learner across training tasks in few-shot learning. It is often assumed that tasks, also called episodes, are sampled from the same distribution $p\left(T\right)$. Each task $T_{i}∼p\left(T\right)$ has both training and test data, denoted as $T_{i}=\left\{D_{i}^{support},D_{i}^{query}\right\}$. Often, the training and test datasets in each task $T_{i}$ are also called *support* and *query* sets, as shown in Figure 2 (bottom). The intuition behind the episodic strategy is that, although training data in each task is limited, the parameters of meta-learner are shared among many tasks. In effect, from a meta-learning perspective, such a strategy could alleviate the sample burden in a single task as if the number of tasks is large enough.

## 4. Methodology

### 4.1. The Overall Framework

**Figure 3:**
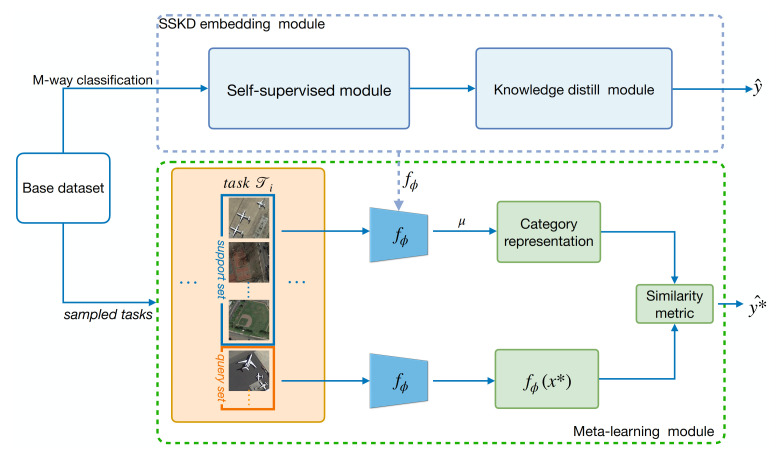
Overall framework of the proposed method. It contains two modules: the SSKD module aims at learning a powerful embedding, without any additional annotation effort that offers more discriminative representations to the downstream meta-learner. The meta-learning module is based on ProtoNets with an additional parameter γ to scale cosine similarity.

As depicted in Figure 3, our model consists of two modules: a self-supervised knowledge distillation (SSKD) embedding module and a meta-learning module. Instead of exploring complex meta-learning structures, we suggest that a good embedding representation might be a powerful boost for achieving or even outperforming SOTA performance on few-shot classification tasks.

**SSKD embedding module** We train the embedding model SSKD on SEEN categories in $D_{base}$ to generate a powerful embedding for the downstream meta learner M(·). The self-supervised learning network and Knowledge distillation procedure are elaborate in Section 4.2 and Section 4.3 and illustrated in Figures 6 and 7. Given a base dataset $D_{base}$ with M (SEEN) categories, we train an M-way classifier on all categories to get the embedding encoder $f_{\phi }$ that mapping the inputs into an embedding space. The embedding encoder $f_{\phi }$ parameterized by ϕ can be optimized by minimizing a loss function $L_{base}$, which will be described in Equation (4), Section 4.2.3.

**Meta-learning module** In this stage, we utilize the downstream meta learner M(·) to optimize the embedding $f_{\phi }$ directly without introducing any extra parameters. To this end, we follow the episodic training manner proposed in [38], which is the most popular meta-learning routine [38,39,40,42,45]. The meta learner is built upon prototypical networks (ProtoNets) [40]. The whole flow of the meta-learning stage is elaborated in Section 4.4.

### 4.2. Learning a Self-Supervised Embedding

In this section, we start by introducing two important components of the proposed self-supervised (SS) network: the backbone ResNet-12 and class activation mapping (CAM). Then, we present the framework of the proposed SS network in detail.

#### 4.2.1. Backbone

**Figure 4:**
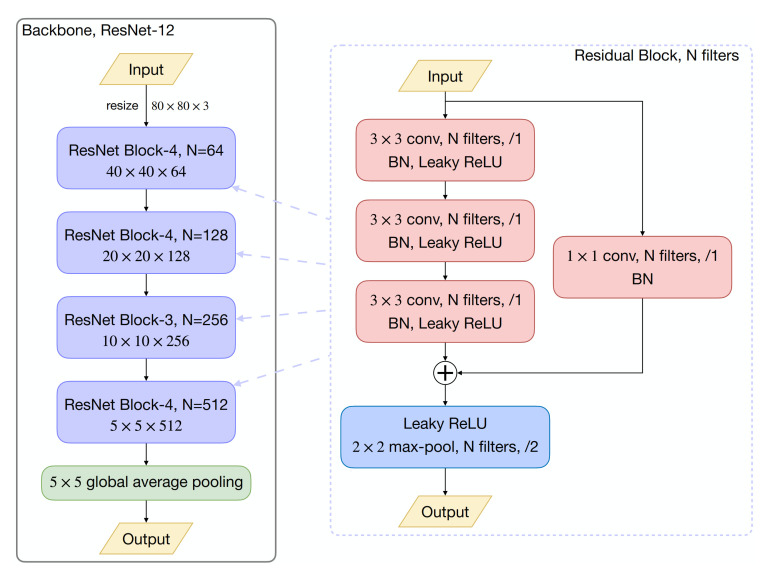
Illustration of the backbone architecture. The left plot shows that ResNet-12 contains four residual blocks, followed by a global average pooling (GAP) layer. Each residual block is a sequential concatenation of three {3×3 convolution with N filters, batch normalization (BN), Leaky ReLU (0.1)}, then a 2×2 max-pooling layer is applied with stride 2, shown in the right plot. The number of filters in each residual block are 64, 128, 256, and 512, respectively.

Most of the early FSL methods [38,40,42,45] utilized a four-layer convolutional network (Conv-4) as the embedding backbone, while more recent models found that such a shallow embedding network might lead to underfitting. In this work, we take ResNet-12, the most popular backbone in current FSL literature [51,52,59], as our embedding network. As illustrated in Figure 4, the ResNet-12 is a smaller version of the ResNet [9], containing four residual blocks and generates 512-dimensional embeddings after a global average pooling (GAP).

#### 4.2.2. Class Activation Mapping

**Figure 5:**
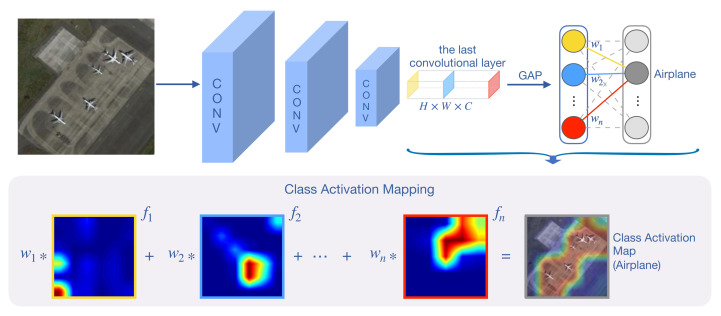
Class Activation Mapping: the predicted classification weight is mapped back to the feature maps generated from the last convolutional layer to compute the class activation maps (CAMs). The CAM highlights the discriminative regions of the corresponding category.

Instead of learning from scratch, we note that Zhang et al. [50] utilize a pre-train stage to classify all SEEN categories with the cross-entropy loss (e.g., 25 categories in the NWPU-RESISC45 Dataset). The feature maps generated by the last residual block are fed to the GAP layer, whose weights are then used as initialization in the meta-training stage. However, we argue that a mixed global representation might lose useful features as the dominant objects can locate anywhere on the image. A technique named *Class Activation Mapping* (CAM) [60] is proposed to tackle this problem; it enables the network to locate the most relevant regions in the image and reduce the distraction caused by irrelevant parts. Consider the ResNet-12 (as well as other typical CNN networks) as the backbone; CAM is depicted in Figure 5. Given an image x, the feature maps generated from the last convolutional layer is denoted as $F\in R^{C\times H\times W}$, where C,H, and *W* are the number of channels, height, and width of the feature maps, respectively. Let $f_{k}\left(x,y\right)$ denote the activation of *k*-th feature map at spatial location (x,y), where k∈{1,…,C}. We perform GAP on the feature maps F, and the pooled features become in size of C×1×1. Then, for $f_{k}$, the corresponding pooled feature is denoted as $F_{k}$. Assume we do an L-way classification here; for a given category *c*, we define $w_{k}^{c}$ as the weight of $F_{k}$ for category *c*. Letting $M_{c}$ be the class activation map for class *c*, we need to only compute the sum

(1) $$M_{c}\left(x,y\right)=\sum_{k=1}^{C}w_{k}^{c}f_{k}\left(x,y\right).$$

That is, $M_{c}\left(x,y\right)$ represents the activation map at spatial grid (x,y) for category *c*. Note that the size of activation map in Equation (1) is H×W, which needs to be upsampled to match the input image size.

#### 4.2.3. Self-Supervised Network

**Figure 6:**
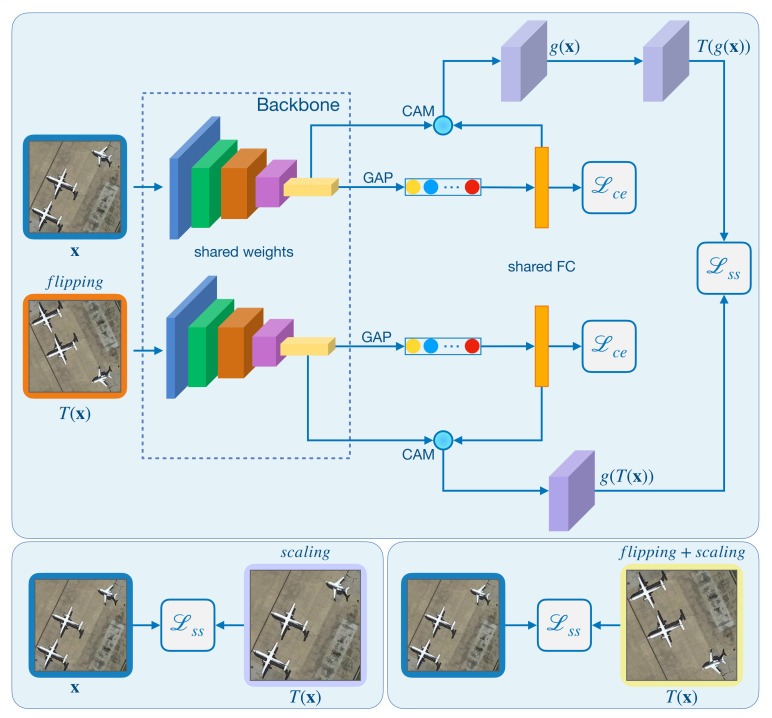
The proposed self-supervised network. We take the original-transformed image pairs as inputs and compute the Class Activation Maps (CAMs) for each input image. Then, we define a new self-supervised loss as a distance between the transformed CAMs of the original image and the CAMs of the transformed image.

Most of the prior works [61,62] in computer vision weave self-supervision into few-shot learning by adding pretext tasks loss. Predicting the index of jigsaw puzzles and the angle of rotations are among the most effective pretext task choices. The most important FSL benchmark in computer vision is miniImageNet [38], whereas RS scene datasets have their characteristics. For example, for an image of a dog, the most discriminative part of the image changes when flipping or rotating it. However, RS scene images may lack this crucial discriminative information because many natural objects (e.g., forest, desert, ocean, and so forth) have fractal properties [63]. Taking this in mind, we propose a novel two-branch network that takes pairs of original-transformed images as inputs and incorporates CAM [60] to force the network to mine the most relevant category-specific region, shown in Figure 6.

Given an original image x, we apply a transformation function T(·) to generate its auxiliary copies of x. Let T denote the set of transformations, and any suitable transformation can be embedded in the proposed self-supervised network. In this work, we consider three transformed copies, applying $\left\{vertical\;flipping\right\}$, $\left\{scaling\right\}$ and $\left\{vertical\;flipping+scaling\right\}$ to x, we create $\left\{T_{f}\left(x\right),T_{s}\left(x\right),T_{fs}\left(x\right)\right\}\in T$ to enhance the feature discriminability for classification.

As shown in Figure 6, we take ResNet-12 as the feature extractor, identical in both branches, and sharing the parameters. The image pairs are fed into backbones, each containing four residual blocks following by a GAP layer and end with an FC (fully connected) layer. The feature maps F are generated from the last residual block and flattened by a GAP player into features with a dimension of 1×1×C. For an original-transformed image pair x and T(x), the corresponding feature maps are $F\in R^{H\times W\times C}$ and $F^{\prime }\in R^{H^{\prime }\times W^{\prime }\times C}$, respectively. Now, the CAMs of input x can be computed by Equation (1). We use M=g(x) to indicate the corresponding CAMs of image x, where g(·) is the procedure of generating CAMs with Class Activation Mapping. Similarly, the resulting CAMs of the transformation T(x) are denoted as $M^{\prime }=g\left(T\left(x\right)\right)$.

Inspired by the study of [64], we transform the CAMs g(x) of the original image into T(g(x)) to enforce visual attention consistency. For example, suppose the inputs are $\left\{x,T(x)\right\}$, where T(x) denotes the original image’s vertical flipping transformation. In that case, we flip the CAMs g(x) of the original image vertically to obtain T(g(x)), and let $M^{T}=T\left(M\right)=T\left(g\left(x\right)\right)$. Then, we design a self-supervised loss as the mean square difference between the transformed CAMs $M^{T}$ of the original image and the CAMs $M^{\prime }$ of the transformed image, which formalized as

(2) $$L_{ss}=\frac{1}{NLHW}\sum_{i=1}^{N}\sum_{j=1}^{L}{∥M_{ij}^{T}-M_{ij}^{\prime }∥}_{2},$$

where $M_{ij}$ represents the CAMs for image *i* and label *j*, and L is the total number of labels. Note that only the flipping copy of x is considered in Equation (2); other transformations such as rotation and scaling can also be embedded in the proposed network. Here, we consider linearly combine three transformed copies, $\left\{T_{f}\left(x\right),T_{s}\left(x\right),and\;T_{fs}\left(x\right)\right\}\in T$, to enforce the attention consistency under certain transformed pairs, which leads the network digging to be the most relevant category-specific region and benefit the classification performance. Thus, our final self-supervised loss can be combined as

(3) $$L_{ss,total}=L_{ss,T_{f}}+L_{ss,T_{s}}+L_{ss,T_{fs}}$$

Let $L_{ce}$ be the cross entropy loss between the predictions and the true labels; our final loss function is then

(4) $$L_{base}=L_{ce}+L_{ss,total}.$$

We now use the combined loss $L_{base}$ to train the network, and the whole procedure of training the model can be cast as the following optimization problem:

(5) $$\phi =\underset{\phi }{\mathrm{argmin}}L_{\mathrm{base}}\left(D_{\mathrm{base}};\phi \right).$$

The above objective ensures that the embedding $f_{\phi }$, parameterized by ϕ, is representative enough to capture the category-specific region information of the input x.

### 4.3. Self-Distillation

**Figure 7:**
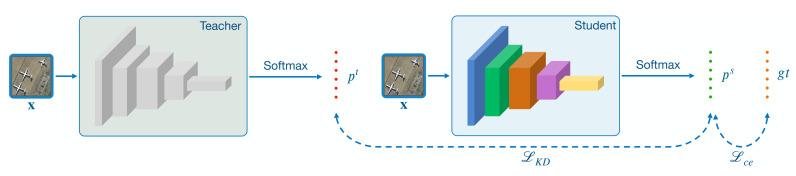
Illustrative diagram of our self-distillation procedure, which boost the performance of the self-supervised module.

Knowledge distillation (KD) [65] is an approach that "knowledge" is transferred from one model (teacher) to another (student). In particular, it is called self-distillation if the teacher and student share identical architecture [66]. The idea of self-distillation is to retrain the predictions of the trained model as new target values and empirically iterate the loop one or several times. The authors of [66] theoretically analyzed that a few self-distillation generations can reduce over-fitting, while further generations may lead to under-fitting and trending worse performance. Inspired by this, we leverage a round of self-distillation to boost the performance of the proposed model. Here, we empirically take only one generation, as we are dealing in the low data regime. We start by constructing two clones of the self-supervised network trained in Section 4.2.3, which, as shown in Figure 7: one serves as a teacher model and another as a student model.

Let $p^{t}$ and $p^{s}$ denote the logits that input x pass through the teacher and the student networks, respectively. We freeze the teacher network’s weights and train the student one by minimizing the combination of two loss function terms. Define $L_{ce}$ as the cross-entropy loss between the student predictions and ground-truth labels, and $L_{KD}$ as the Kullback–Leibler divergence (KL) loss between the teacher and the student predictions. Then, the combined loss function is:

(6) $$\phi^{\prime }=\underset{\phi^{\prime }}{\mathrm{argmin}}\left(L_{ce}\left(D_{\mathrm{base}\;};\phi^{\prime }\right)\right)+KL\left(f\left(D_{\mathrm{base}\;};\phi^{\prime }\right),f\left(D_{\mathrm{base}\;};\phi \right)\right),$$

where ϕ and $\phi^{\prime }$ represent the parameters of the teacher and the student network, respectively. We use the distilled network $f_{\phi }$ as the final embedding model to extract features for meta-training.

### 4.4. Meta-Learning Module

In the standard few-shot learning field, models are often training and evaluated in *N*-way *K*-shot tasks. As defined in the literature [38], an *N*-way *K*-shot task in the meta-learning stage is constructed as follows. *N* different categories are randomly sampled from the set of SEEN categories for each task, then *K* *support* examples in each of the *N* categories are selected for training. Simultaneously, *Q* *query* instances are randomly selected from the remaining of that category to be classified. A set of tasks $\left\{T_{i}\right\}$ drawn from SEEN categories $C_{base}$ is referred to as a meta-training set $T^{train}$. In the same manner, we can form a meta-validation set $T^{val}$ from $C_{val}$ and a meta-test set $T^{test}$ from $C_{novel}$. Given the training data $T^{train}$, we adopt a meta-learning routine similar to Prototypical Networks (ProtoNet) [40], and the embedding model $f_{\phi }$ is optimized by minimizing the generalization error across tasks. The learning objective can be formalized as:

(7) $$\phi =\underset{\phi }{\mathrm{argmin}}E_{T^{\mathrm{train}\;}}\left[L_{\mathrm{meta}\;}\left(D^{\mathrm{query}\;};\phi \right)\right].$$

For an *N*-way *K*-shot task, $T_{i}$ with the support set $D_{i}^{\mathrm{support}\;}={\left\{x_{k},y_{k}\right\}}_{k=1}^{NK}$, where $y_{k}\in \left\{1,\ldots ,N\right\}$; each training sample $x_{k}$ is mapping to $f_{\phi }\left(x_{k}\right)$. ProtoNet computes the mean feature of *K* support samples belonging to category *c* as its "prototype":

(8) $$p_{c}=\frac{1}{K}\sum_{y_{k}=c}f_{\phi }\left(x_{k}\right),\forall c=1,\ldots ,N$$

To classify a test sample $x_{q}$ in the query set, we build a cosine similarity based classifier. The probability of using the softmax function to predict the query $x_{q}$ as class *c* is

(9) $$p\left(y_{q}=c|x_{q}\right)=\frac{\exp\left(\gamma \cdot \cos\left(f_{\phi }\left(x_{q}\right),p_{c}\right)\right)}{\sum_{c^{\prime }=1}^{N}\exp\left(\gamma \cdot \cos\left(f_{\phi }\left(x_{q}\right),p_{c^{\prime }}\right)\right)},$$

where γ is a temperature parameter [51] over the similarity score. We observed that the cosine similarity metric works well with a large temperature value; γ is empirically set to 10. Note that, once the meta-learning phase is done, the embedding model $f_{\phi }$ parameterized by ϕ, is fixed; we do not fine-tune in the meta-test stage.

## 5. Experiment and Results

We first describe the datasets in Section 5.1. The implementation details used in our experiments are presented in Section 5.2. In Section 5.3, we proceed to compare the proposed RS-SSKD method with the state-of-the-art FSL methods on two challenging RS datasets: NWPU-RESISC45 [22] and RSD46-WHU [34]. Finally, in Section 5.4, we conduct an ablation study to investigate the effect of each component in SSKD module.

### 5.1. Datasets

**Figure 8:**
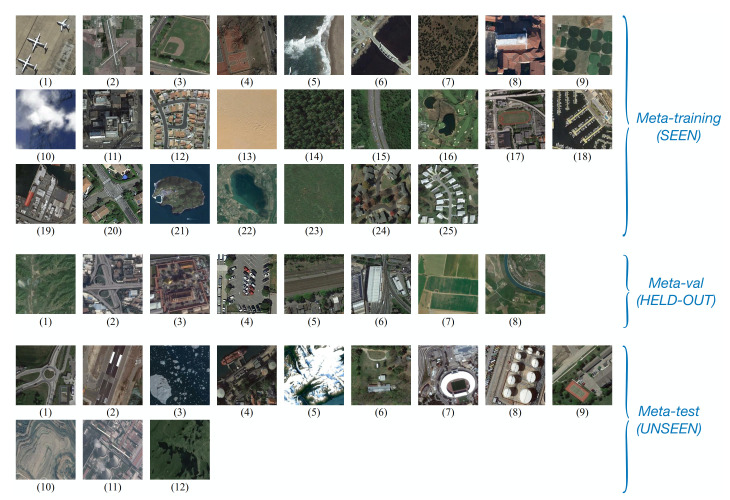
NWPU-RESISC45 Dataset split. Meta-training (SEEN): (1) airplane, (2) airport, (3) baseball diamond, (4) basketball court, (5) beach, (6) bridge, (7) chaparral, (8) church, (9) circular farmland, (10) cloud, (11) commercial area, (12) dense residential, (13) desert, (14) forest, (15) freeway, (16) golf course, (17) ground track field, (18) harbor, (19) industrial area, (20) intersection, (21) island, (22) lake, (23) meadow, (24) medium residential, (25) mobile home park; Meta-validation (HELD-OUT): (1) mountain, (2) overpass, (3) palace, (4) parking lot, (5) railway, (6) railway station, (7) rectangular farmland, (8) river; Meta-test (UNSEEN): (1) roundabout, (2) runway, (3) sea ice, (4) ship, (5) snowberg, (6) sparse residential, (7) stadium, (8) storage tank, (9) tennis court, (10) terrace, (11) thermal power station, (12) wetland.

In the NWPU-RESISC45 dataset, there are 45 categories, each of which has 700 images with a size of 256×256 pixels. This dataset was proposed in 2017 by Cheng et al. [22]; the RS scene images in it are extracted from Google Earth by experienced experts. The spatial resolution of most scene categories ranges from about 30 to 0.2 m per pixel, except for some categories that have lower spatial resolutions, e.g., island, lake, mountain, and snow-berg. Following the split setting proposed by Ravi et al. [39], we take 25 of 45 categories for meta-training, 8 for validation, and 12 for meta-test, as detailed in Figure 8. Namely, a model is trained on many N-way K-shot tasks sampled from the 25 SEEN categories during the meta-training stage. The best model is chosen based on the few-shot classification performance over eight HELD-OUT categories of Meta-val. This best model is our final model, which is tested on the UNSEEN set Meta-test.

**Figure 9:**
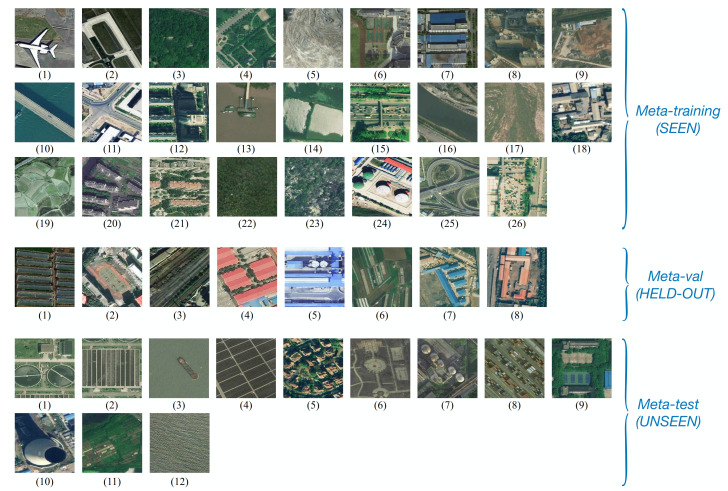
RSD46-WHU Dataset split. Meta-training (SEEN): (1) Airplane, (2) Airport, (3) Artificial dense forest land, (4) Artificial sparse forest land, (5) Bare land, (6) Basketball court, (7) Blue structured factory building, (8) Building, (9) Construction site, (10) Cross river bridge, (11) Crossroads, (12) Dense tall building, (13) Dock, (14) Fish pond, (15) Footbridge, (16) Graff, (17) Grassland, (18) Low scattered building, (19) Lrregular farmland, (20) Medium density scattered building, (21) Medium density structured building, (22) Natural dense forest land, (23) Natural sparse forest land, (24) Oiltank, (25) Overpass, (26) Parking lot; Meta-validation (HELD-OUT): (1) Plasticgreenhouse, (2) Playground, (3) Railway, (4) Red structured factory building, (5) Refinery, (6) Regular farmland, (7) Scattered blue roof factory building, (8) Scattered red roof factory building; Meta-test (UNSEEN): (1) Sewage plant-type-one, (2) Sewage plant-type-two, (3) Ship, (4) Solar power station, (5) Sparse residential area, (6) Square, (7) Steelsmelter, (8) Storage land, (9) Tennis court, (10) Thermal power plant, (11) Vegetable plot, (12) Water.

The RSD46-WHU dataset contains 117,000 images of RS scenes over 46 categories, with around 500–3000 images in each. These images are gathered from Google Earth and Tianditu by hand, and the ground resolution is 0.5 m for most categories while about 2 m for others. Similar to the configuration of the NWPU-RESISC45 dataset, we divide it into 26, 8, and 12 categories for meta training, validation, and test, respectively. The dataset-split is shown in Figure 9.

### 5.2. Implementation Details

**Backbone.** Following recent works [50,51,52,59], we use Resnet-12 [9] as the backbone in both the SSKD module and the meta-learning module. A GAP layer is added to the last ResNet Block, which outputs 512-dimensional embeddings; the details are introduced in Section 4.2.1. All inputs are resized to 80×80×3, except the scaling transformations in the self-supervised network, resize to 96×96×3. Here, we interpolate the resized 80×80 images to 96×96 instead of resizing directly with the original 256×256 images, as most of the FSL literature takes images of size 80×80 or 84×84 as inputs. For this case, we apply a 6×6 GAP, which generates 512-dimensional embeddings, likewise.

**Optimization.** For the SSKD module, we adopt SGD with a momentum of 0.9 and weight decay of 0.0005. The learning rate is set to 0.1 at the beginning and decays at epoch 90, and the decay factor is 0.1. We train 110 epochs with batch size 64 on both datasets. For the meta-learning module, SGD is used with a fixed learning rate of 0.001, weight decay of 0.0005. We set four tasks in a batch to compute the average loss; namely, the batch size is 4. An epoch contains 200 batches, that is, 800 tasks. We empirically meta-train the model for 90 epochs. In our case, the best model often occurs in the first 60 epochs. Note that, in conventional machine learning, one epoch refers to pass all the training data forward and backward through the network once. In few-shot learning, tasks are randomly sampled from the dataset. Though the support (training) data in each task are limited, we can assume that the entire dataset has probably been traversed when an epoch holds enough tasks. Pytorch is applied to implement all our experiments on four NVIDIA RTX 3090 GPUs.

### 5.3. Main Results

**Figure 10:**
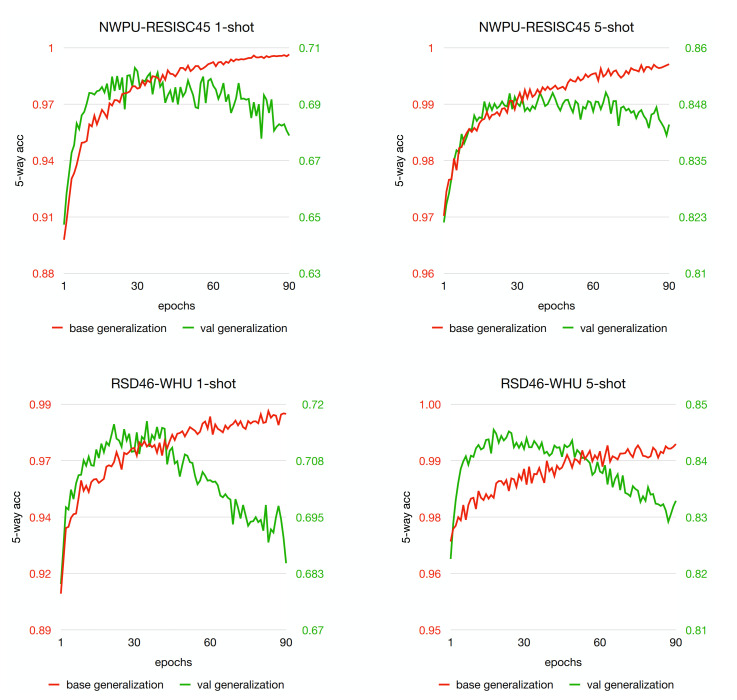
Generalization curves of the proposed method on meta-train and meta-val sets with respect to the number of training epochs. The 5-way classification accuracy with 1-shot and 5-shot at each epoch is reported.

We verify the effectiveness of the proposed RS-SSKD method on two datasets, NWPU-RESISC45 [22] and RSD46-WHU [34]. The same evaluation protocol is used over all the experiments. Following the prior work [38,39,40], the tasks between meta-training, meta-validation, and meta-test should be in the same configuration. For example, in the 5-way 1-shot scenario, a task includes five categories (way), each category with only one support samples (training data), along with 15 query samples to be classified (test data). We keep sampling the 5-way 1-shot tasks from SEEN categories during meta-training. The tasks drawn from the meta-val categories (HELD-OUT) are used for selecting the best model. The model generalization accuracy in the meta-learning stage is shown in Figure 10, where the best model may appear at the epoch corresponding to the peak of the green line. Once the meta learning stage is done, the best model is applied to those tasks sampled from meta-test (UNSEEN) categories for evaluation. The same protocol is used for the 5-way 5-shot case. Note that, in the standard FSL setting, the meta-test tasks arrive one at a time, not simultaneously. In other words, we record the accuracy when every task comes and compute the mean accuracy over many tasks, with a 95% confidence interval.

Table 2 and Table 3 show the results of several FSL approaches on the NWPU-RESISC45 dataset, where both 5-way 1-shot and 5-way 5-shot classification performance are reported. The main results of comparison approaches are cited from a recent study [50] on few-shot classification of RS scenes. The methods marked with an asterisk indicate that the backbone of the original method is replaced with Resnet-12. Most of the comparison methods evaluate the models on 600 tasks sampled from the UNSEEN categories, which leads to high variance. We follow the more reliable evaluation setting suggested by Zhang et al. [50], evaluating our method on 8000 sampled tasks. The mean accuracy (in %) with 95% confidence interval is reported for comparison. On both datasets, our RS-SSKD approach outperforms the previous results.

**Figure 11:**
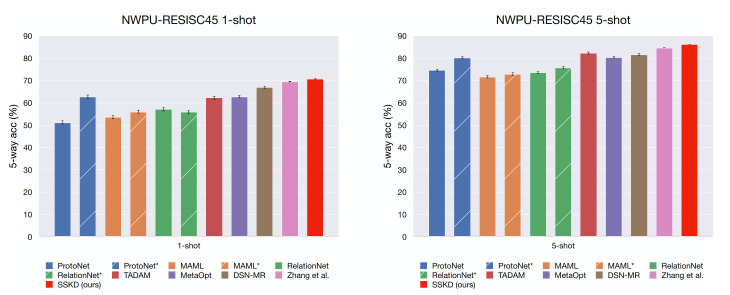
The few-shot classification performance (with 95% confidence intervals) for the NWPU-RESISC45 dataset, the striped bars indicate the re-implementation of approaches with a Resnet-12 backbone.

**Figure 12:**
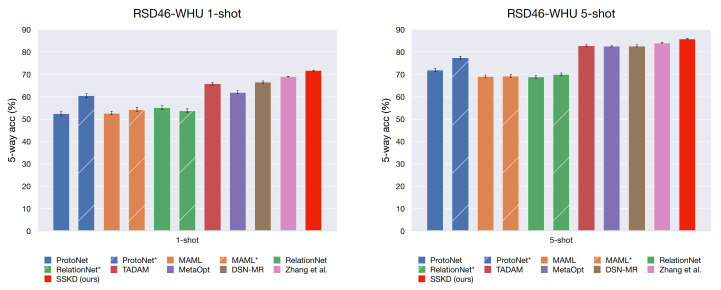
The few-shot classification performance (with 95% confidence intervals) on the RSD46-WHU dataset, the striped bars indicate the re-implementation of approaches with a Resnet-12 backbone.

To more clearly observing whether the backbone impacts the performance, we plot bar charts in Figure 11 and Figure 12, and the striped bars indicate the re-implementation of approaches with the Resnet-12 backbone. It is surprising that the re-implementation of MAML [42] only gets minor improvements with Resnet-12 over Conv4 while RelationNet [45] even drops in the 5-way 1-shot scenario for both datasets. This phenomenon might occur due to the complex comparison module of RalationNet leads overfitting when leveraging deeper networks. In contrast, ProtoNet [40] gets significant improvements when replacing the backbone with Resnet-12. The re-implemented ProtoNet achieves comparable performance to the recent leading approach MetaOpt [52] on the NWPU dataset, known as a powerful approach. DSN-MR [59] is based on ProtoNet by mapping the mean category feature and query samples into a subspace, and performing a distance metric comparison in the subspace. It achieves good few-shot classification performance while consumes a lot of computational resources, e.g., at least four GPUs with ∼10GB/GPU are required to train the model. TADAM [51] proposed a dynamic feature extractor that can be optimized in a task-conditioned manner, yet extra parameters and additional complexity are carried to the network. They solve this issue by utilizing an additional logit head (i.e., the normal M-way classification, where M is the category number in the base set) for co-training. The authors claim that such a strategy on miniImageNet [38] is better than simple pre-training; however, we observe the opposite result in both RS scene datasets, NWPU-RESISC45 [22] and RSD46-WHU [34].

**Figure 13:**
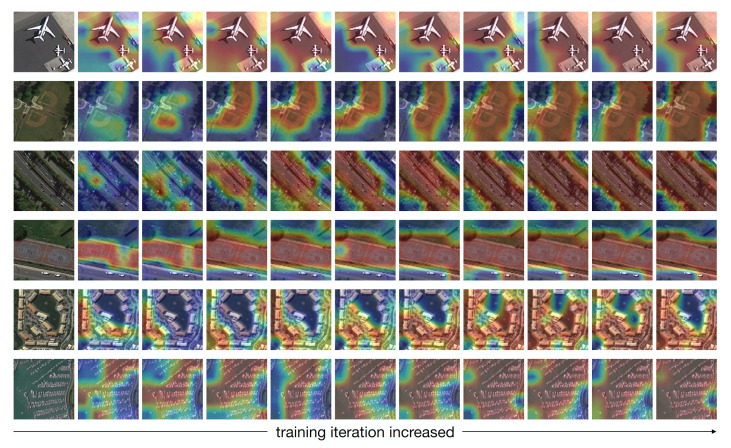
The class activation maps (CAMs) of images training by our self-supervised network, where the corresponding labels are: airplane, baseball diamond, freeway, basketball court, dense residential, and harbor. The training iteration increased from **left** to **right**.

The leading method [50] employs a plain pre-training head over the SEEN categories, based on which the model is further optimized in the meta-training stage. The major difference between method [50] and ours is that the former trains the backbone network to classify all SEEN categories with the cross-entropy loss (e.g., 25 categories in the NWPU-RESISC45), and adds a global average pooling layer to reduce the dimension of the embedding. However, we argue that some useful features might be lost by the mixed global representation as the dominant objects could locate anywhere on the image. This point is especially challenging in RS scenes as the within-class diversity and between-class similarity are still two big problems. For example, given a freeway image, does the network focus on the freeway or the forest on the sides of the freeway? Intuitively, we expect the network to dig the most discriminative and transferable features that might be important cues for image classification, especially in a low-data regime. Our SSKD module addresses this problem by incorporating the CAMs into the proposed two-branch self-supervised network, enabling the network to discover the most relevant regions in the image and reduce the distraction caused by irrelevant parts. This idea is inspired by human attention behavior. If the network is able to highlight the regions that are semantically relevant to the correspondence labels, one can expect better classification performance. As illustrated in Figure 13, we can observe that the proposed network captures the most relevant regions to the corresponding categories. The results in Table 2 and Table 3 suggest that our method consistently surpasses the work [50] (similar to our meta-learning stage) on both datasets. This verifies that our SSKD module learns a discriminative embedding, thus improving the representation capabilities of our model.

### 5.4. Ablation Studies and Discussion

In this section, we give a visualization analysis of the proposed self-supervised network at first. Then, SSKD-module and its ablated variants are analyzed on the NWPU-RESISC45 and RSD46-WHU datasets with ResNet12 backbone. Finally, we analyze the training time of state-of-the-art methods and ours.

#### 5.4.1. Visualization Analysis

**Figure 14:**
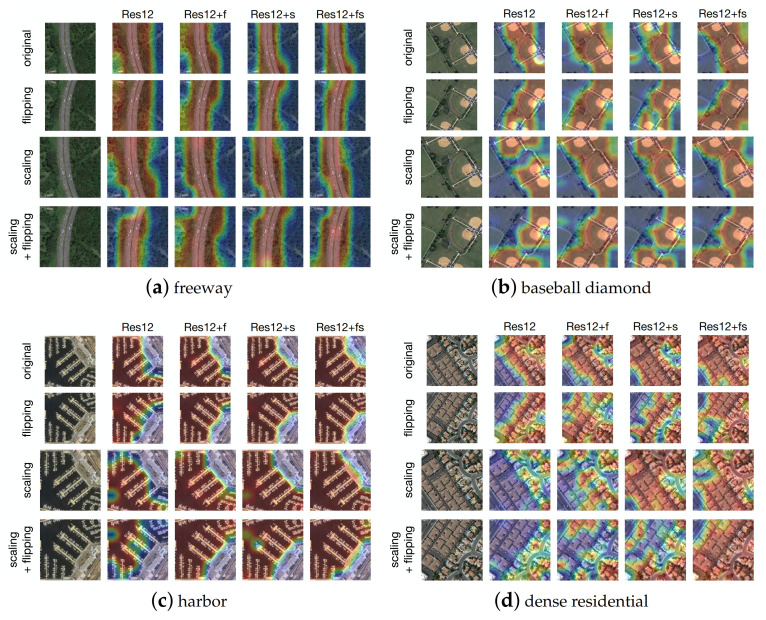
Class activation maps (Cams) visualization according to the labels: (**a**) freeway; (**b**) baseball diamond; (**c**) harbor; and (**d**) dense residential using different models.

To verify that our proposed self-supervised network refines the class activation maps (CAMs), we compare the visualization results generated from the original, flipped, and scaled inputs for the same label using the baseline model (Resnet-12) and the proposed network. Using the proposed self-supervised network that enforces the attention consistency (AC) [64] under the three image transformed pairs, we get the trained models: Res12+f (flipping), Res12+s (scaling), and Res12+fs (flipping and scaling), respectively. Figure 14 shows four examples of CAMs for the labels (a) freeway; (b) baseball diamond; (c) harbor; and (d) dense residential. The CAMs from the baseline model Resnet-12 are either inconsistent or contain non-target parts, e.g., it covers a lot of the forests on both sides of the freeway under both the vertical flipping and scaling transformations. Our Res12+f model generates highly consistent attention regions under image flipping but failed under image scaling and flippling+scaling. The Res12+s model produces highly inconsistent CAMs under image flipping and scaling+flilping, especially in the cases (b) baseball diamond and (d) dense residential. Considering attention consistency under both flipping and scaling, the model Res12+fs outputs highly consistent CAMs under all cases except slightly inconsistent under image scaling and flipping in (d), but still better than others. These qualitative results illustrate that the proposed network can generate the most relevant category-specific regions by enforcing attention consistency under original-transformed pairs.

#### 5.4.2. The Effect of Auxiliary Loss Functions

Here, we empirically show the contributions of each auxiliary loss by progressively incorporating them into the proposed SSKD module. To this end, we start with plain cross-entropy as our baseline, denoted as $L_{ce}$. Here, we use the abbreviated $L_{ss}$ to indicate the final combined self-supervised loss in Equation (3). From the results in Table 4, we observe that the classification performance slightly increased when performing the knowledge distillation (KD) loss on models trained on $L_{ce}$. Then, if we train the model with the proposed self-supervised network, the model performance improves to 69.72% and 84.87% on the NWPU-RESISC45 dataset, for 5-way 1-shot and 5-way 5-shot, respectively. On the RSD46-WHU dataset, the self-supervised loss gives an absolute gain of 1.61% and 1.48% to the classification performance of 1-shot and 5-shot, respectively. The last row of Table 4 indicates the model trained with the whole SSKD module. We can see that, compared with the model trained with plain $L_{ce}$, $L_{KD}$ loss provides more benefits to the model trained with the self-supervised network. These empirical evaluations clearly demonstrate the individual importance of different components in the proposed module.

#### 5.4.3. Training Time Analysis

We report the meta-training runtime of state-of-the-art methods and ours on both datasets, NWPU-RESISC45 [22] and RSD46-WHU [34], in Table 5. The Conv-4 architectures of ProtoNet [40], MAML [42], and RelationNet [45] are 64-64-64-64, 32-32-32-32 64-96-128-256, respectively; the number indicates the filters per layer, as in the original literature. We use the Adam optimizer with an initial learning rate of 0.001 for Conv-4, as suggested in [67]. The ResNet-12 backbone for ProtoNet, MAML, and RelationNet are the same as ours, see Section 4.2.1. MAML states that using 64 filters or a deeper backbone may cause overfitting; to avoid this, we apply standard data augmentation, including random crop, left-right flip, and color jitter, to our implementation of MAML with ResNet-12 backbone. The same data augmentation is applied to ProtoNet and RelationNet to ensure a fair comparison. The hyperparameters of all the methods are following their original settings, e.g., TADAM [51] suggests 30,000 tasks/episodes for meta-training while DSN-MR [59] sets 80,000. The number of tasks in each epoch of these methods is varied, e.g., the early FSL methods like ProtoNet and RelationNet set each epoch with 100 tasks and meta-train for 600 epochs. The more recent work like MetaOpt [52] and DSN-MR [59] were meta-trained for 60 and 80 epochs, respectively, with each epoch consisting of 1000 tasks. Table 5 shows the running time over total meta-training tasks; all models are evaluated on a single GPU RTX 3090, except DSN-MR, which needs at least two RTX 3090 GPUs, due to the high GPU memory consumption of the SVD step.

As shown in Table 5, we observe that the training time for both ProtoNet and RelationNet increases slightly for the ResNet-12 version compared to Conv-4. In comparison, the runtime of MAML with Resnet-12 backbone takes more than two times longer to train than Conv-4. While nearest-neighbor classifier and its variants [40,45,50] are popular in FSL as the classification rule is simple, MetaOpt [52] argues that discriminatively trained linear classifiers often perform better than nearest neighbor classifiers in low data scenarios as they can learn better category boundaries. By incorporating a differentiable quadratic programming (QP) solver [68], MetaOpt proposed an end-to-end model that learns the embedding with various linear classifiers for few-shot classification. It achieves a good performance on both datasets with a significant increase in training time. The runtime of DSN-MR [59] is very slow due to the computational cost in the SVD step; adopting a fast approximate SVD algorithm such as [69] might reduce the training time.

In addition to the training time of the meta-training stage, like Zhang et al. [50], our method has an additional training time for the pre-training stage, which is the runtime of our SSKD module. The pre-training runtime of [50] is 1.6 h on NWPU-RESISC45 and 2.2 h on RSD46-WHU. The training time of our SSKD module is including two parts, the runtime for the self-supervised network and the KD procedure. It cost 3.9 h and 6.3 h to train the proposed self-supervised network on NWPU-RESISC45 and RSD46-WHU, while the KD procedure takes 2.5 h on NWPU-RESISC45 and 2.8 h on RSD46-WHU, respectively. That is, the training time of our SSKD module is 6.4 h and 9.1 h in total on NWPU-RESISC45 and RSD46-WHU, respectively. The same as in work [50], we do not introduce any extra parameters but optimize the embedding directly by the cosine classifiers over the N-way K-shot tasks in the meta-learning stage. Our meta-training runtime is nearly the same as work [50], slightly more than ProtoNet and RelationNet with a ResNet-12 backbone. Counting in the training time of the SSKD module, our approach achieves the best performance at a modest increase in total training time. Note that our SSKD module only needs to train once on a dataset to provide a powerful embedding for downstream meta-learners under arbitrary N-way K-shot setting.

An interesting phenomenon we observed is that, for almost all methods, the meta-training runtime is virtually the same on both datasets. The reason is that, for most methods, the training time for the meta-learning stage depends on the total number of training tasks/episodes, and this number is the same for both datasets. In our experiments, the only exception is TADAM [51], which incorporates a co-training strategy in the meta-learning stage. This strategy introduces additional complexity to the model by adopting an additional logit head (i.e., the normal M-way classification, where M is the number of all SEEN categories) for auxiliary co-training. The co-training burden consumes more training time since the RSD46-WHU dataset is larger than the NWPU-RESISC4 dataset.

## 6. Conclusions

While there is no doubt that the meta-learning procedure plays a significant role in generalization when facing a scarce data regime, from the learning to learn perspective, it makes no sense to meta-learn from scratch. To this end, we proposed a SSKD module: one aims to learn a powerful embedding, without any additional annotation effort that offers more efficient and effective representations to downstream meta-learners. Firstly, a two-branch self-supervised network is designed to catch the most relevant category-specific region of inputs, which forces the network to output more discriminative embeddings. Secondly, we adopt a self-distillation procedure to prevent overfitting and improve the classification performance. Extensive experiments are conducted on two RS scene datasets, and the results verified the effectiveness of the proposed method by achieving the SOTA performances. While our experiment results are very encouraging, they are not enough from a practical standpoint. Much can be done toward the goal of human-level performance. Our future work may include improving the meta-learning process by learning to learn the network backbones and investigate more real applications.

## Figures and Tables

**Table 1:** Publicly available RS scene datasets.

Dataset	# of Categories	Images per Category	Total Images	Image Sizes	Year
UC Merced dataset [16]	21	100	2100	256×256	2010
WHU-RS19 [27]	19	50	950	600×600	2010
AID dataset [32]	30	220–420	10,000	600×600	2016
NWPU-RESISC45 [22]	45	700	31,500	256×256	2016
RSD46-WHU [34,35]	46	500–3000	117,000	256×256	2016
PatternNet dataset [33]	38	800	30,400	256×256	2017

**Table 2:** Comparison to prior works on NWPU-RESISC45. Average 5-way accuracy (%) is reported with a 95% confidence interval. * represents the backbone of the original method is replaced with Resnet-12. Values in bold indicate the ones with the highest classification accuracy.

Method	Backbone	1-Shot	5-Shot
ProtoNet [40]	Conv4	51.17 ± 0.79	74.58 ± 0.56
ProtoNet *	ResNet12	62.78 ± 0.85	80.19 ± 0.52
MAML [42]	Conv4	53.52 ± 0.83	71.69 ± 0.63
MAML *	ResNet12	56.01 ± 0.87	72.94 ± 0.63
RelationNet [45]	Conv4	57.10 ± 0.89	73.55 ± 0.56
RelationNet *	ResNet12	55.84 ± 0.88	75.78 ± 0.57
TADAM [51]	ResNet12	62.25 ± 0.79	82.36 ± 0.54
MetaOpt [52]	ResNet12	62.72 ± 0.64	80.41 ± 0.41
DSN-MR [59]	ResNet12	66.93 ± 0.51	81.67 ± 0.49
Zhang et al. [50]	ResNet12	69.46 ± 0.22	84.66 ± 0.12
RS-SSKD (ours)	ResNet12	**70.64 ± 0.22**	**86.26 ± 0.12**

**Table 3:** Comparison to prior works on RSD46-WHU. Average 5-way accuracy (%) is reported with 95% confidence interval. * represents the backbone of the original method is replaced with Resnet-12. Values in bold indicate the ones with the highest classification accuracy.

Method	Backbone	1-Shot	5-Shot
ProtoNet [40]	Conv4	52.57 ± 0.89	71.95 ± 0.71
ProtoNet *	ResNet12	60.53 ± 0.99	77.53 ± 0.73
MAML [42]	Conv4	52.73 ± 0.91	69.18 ± 0.73
MAML *	ResNet12	54.36 ± 1.04	69.28 ± 0.81
RelationNet [45]	Conv4	55.18 ± 0.90	68.86 ± 0.71
RelationNet *	ResNet12	53.73 ± 0.95	69.98 ± 0.74
TADAM [51]	ResNet12	65.84 ± 0.67	82.79 ± 0.58
MetaOpt [52]	ResNet12	62.05 ± 0.76	82.60 ± 0.46
DSN-MR [59]	ResNet12	66.53 ± 0.70	82.74 ± 0.54
Zhang et al. [50]	ResNet12	69.08 ± 0.25	84.10 ± 0.15
RS-SSKD (ours)	ResNet12	**71.73 ± 0.25**	**85.90 ± 0.15**

**Table 4:** Few-shot classification results on NWPU-RESISC45 and RSD46-WHU, with different auxiliary loss functions.

Loss Function	NWPU-RESISC45, 5-Way		RSD46-WHU, 5-Way	
	1-Shot	5-Shot	1-Shot	5-Shot
$L_{ce}$	69.46 ± 0.22	84.66 ± 0.12	69.08 ± 0.25	84.10 ± 0.15
$L_{ce}\to L_{KD}$	69.72 ± 0.22	84.87 ± 0.12	69.39 ± 0.25	84.31 ± 0.15
$L_{ce}+L_{ss}$	69.91 ± 0.22	85.39 ± 0.13	70.69 ± 0.25	85.58 ± 0.15
$L_{ce}+L_{ss}\to L_{KD}$	70.64 ± 0.22	86.26 ± 0.12	71.73 ± 0.25	85.90 ± 0.15

**Table 5:** Meta-training runtime comparison of methods on NWPU-RESISC45 and RSD46-WHU datasets, under 5-way 1-shot and 5-way 5-shot classification scenarios. * represents the backbone of the original method is replaced with Resnet-12.

Method	Backbone	Meta Training Tasks	NWPU-RESISC45		RSD46-WHU	
			1-Shot Runtime	5-Shot Runtime	1-Shot Runtime	5-Shot Runtime
ProtoNet [40]	Conv4	60,000	1.2h	1.4h	1.2h	1.4h
ProtoNet *	ResNet12	60,000	1.8h	1.9h	1.8h	1.9h
MAML [42]	Conv4	60,000	7.7h	8.3h	7.8h	8.3h
MAML *	ResNet12	60,000	18.2h	19.5h	18h	19.5h
RelationNet [45]	Conv4	60,000	1.4h	1.8h	1.4h	1.7h
RelationNet *	ResNet12	60,000	2.2h	2.5h	2.2h	2.3h
TADAM [51]	ResNet12	30,000	5.9h	7.5h	7.4h	9.5h
MetaOpt [52]	ResNet12	60,000	6.4h	10.2h	6.3h	10.1h
DSN-MR [59]	ResNet12	80,000	33.2h	70.3h	32.9h	70.5h
Zhang et al. [50]	ResNet12	48,000	2.3h	2.9h	2.3h	2.9h
SSKD (ours)	ResNet12	48,000	2.3h	2.9h	2.3h	3.0h

## Data Availability

The datasets involved in this paper are all public datasets and have been appropriately cited.
